# The European Bioinformatics Institute in 2017: data coordination and integration

**DOI:** 10.1093/nar/gkx1154

**Published:** 2017-11-25

**Authors:** Charles E Cook, Mary T Bergman, Guy Cochrane, Rolf Apweiler, Ewan Birney

**Affiliations:** European Molecular Biology Laboratory, European Bioinformatics Institute (EMBL-EBI), Wellcome Genome Campus, Hinxton, Cambridge CB10 1SD, UK

## Abstract

The European Bioinformatics Institute (EMBL-EBI) supports life-science research throughout the world by providing open data, open-source software and analytical tools, and technical infrastructure (https://www.ebi.ac.uk). We accommodate an increasingly diverse range of data types and integrate them, so that biologists in all disciplines can explore life in ever-increasing detail. We maintain over 40 data resources, many of which are run collaboratively with partners in 16 countries (https://www.ebi.ac.uk/services). Submissions continue to increase exponentially: our data storage has doubled in less than two years to 120 petabytes. Recent advances in cellular imaging and single-cell sequencing techniques are generating a vast amount of high-dimensional data, bringing to light new cell types and new perspectives on anatomy. Accordingly, one of our main focus areas is integrating high-quality information from bioimaging, biobanking and other types of molecular data. This is reflected in our deep involvement in Open Targets, stewarding of plant phenotyping standards (MIAPPE) and partnership in the Human Cell Atlas data coordination platform, as well as the 2017 launch of the Omics Discovery Index. This update gives a birds-eye view of EMBL-EBI’s approach to data integration and service development as genomics begins to enter the clinic.

## INTRODUCTION

EMBL’s European Bioinformatics Institute (EMBL-EBI) is a publicly funded scientific organization that provides open data, open-source software tools and ‘big data’ infrastructure to the global life-science research community, free of charge. The institute’s approach to managing, developing and extending these public data resources is influenced by rapid technological advances in the life sciences, such as single-cell sequencing and bioimaging.

Data standards and ontology development are basic components of our integration activities, extending well beyond molecular biology to encompass imaging, biobanking and plant phenotyping. As data generation intensifies across the biomedical and life sciences, analytical pipelines designed for lower data volumes are suffering from bottlenecks. The adoption of data standards can relieve some of this pressure, allowing information to flow into analysis pipelines across sectors.

The speed of mass data production and deposition demands creative solutions for data storage and computational infrastructure. The results of large-scale metagenomics, proteomics and metabolomics experiments are beginning to enter the public archives at scale, and bioimaging is gaining a firm foothold on the data-growth ladder. Analyzing such data collectively puts a strain on local compute infrastructure, and is greatly facilitated by cloud-based collaboration platforms such as those being built by EMBL-EBI.

This update gives an overview of data growth, diversification, integration and analysis in the life sciences at a time of great transformation in the use of data in healthcare, agriculture and biotechnology.

## EMBL-EBI DATA RESOURCES IN 2017

**Figure 1.**
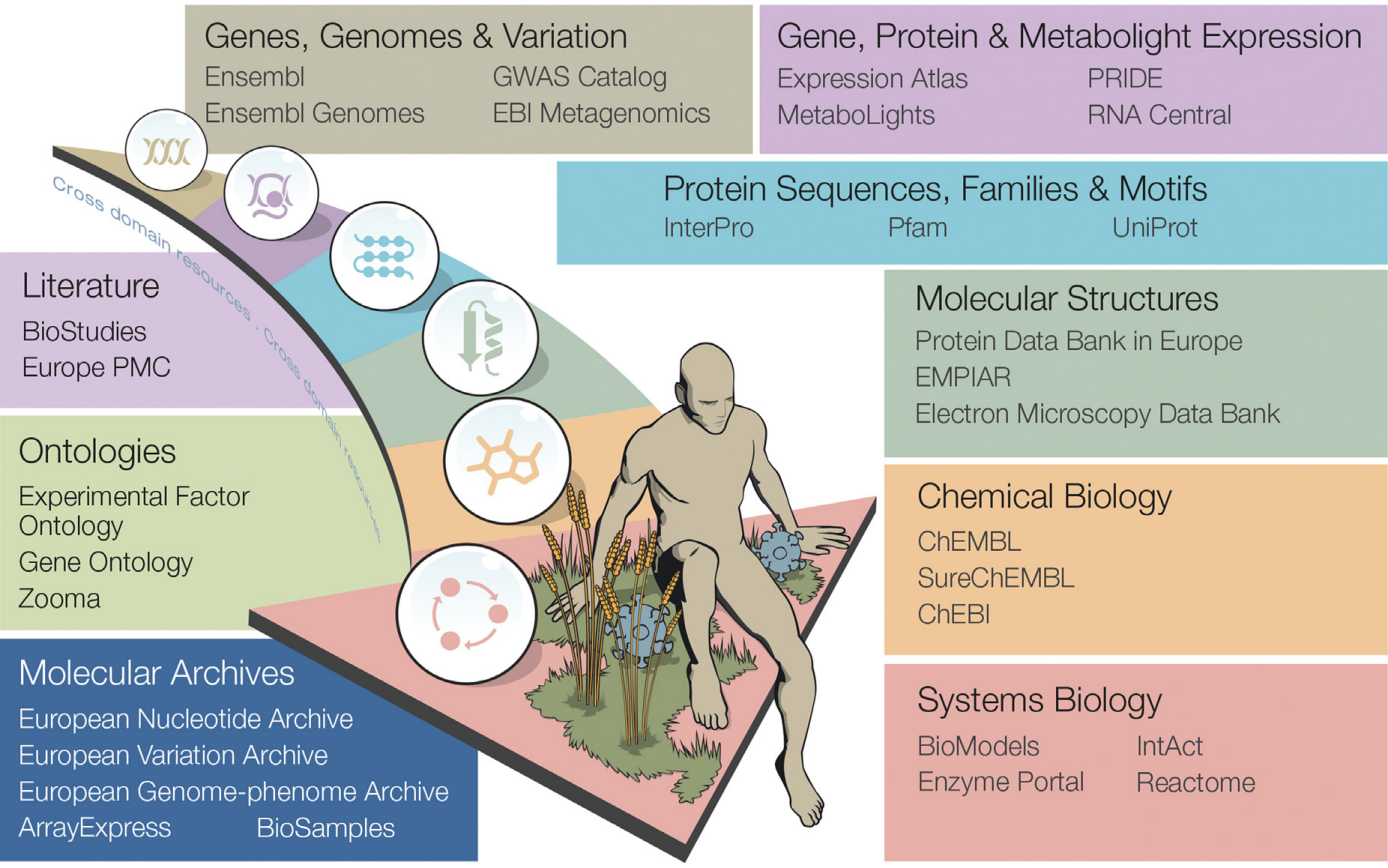
Selection of core data resources at EMBL-EBI. Updates in this issue: BioModels (15), EBI Metagenomics (39), ENA (6), Ensembl (7), Ensembl Genomes (8), Europe PMC (9), Expression Atlas (21), Mechanism and Catalytic Site Atlas (M-CSA) (45), MEROPS (46), Protein Data Bank in Europe (PDBe) (12), Reactome (47), Rfam (48) and WormBase (49). For a complete lists of EMBL-EBI resources see https://www.ebi.ac.uk/services.

The public data resources at EMBL-EBI have become an essential component of any computational biology research project (www.beagrie.com/static/resource/EBI-impact-report.pdf). Updates on several of these resources are featured in this issue of *Nucleic Acids Research*, as shown in Figure 1.

**Figure 2.**
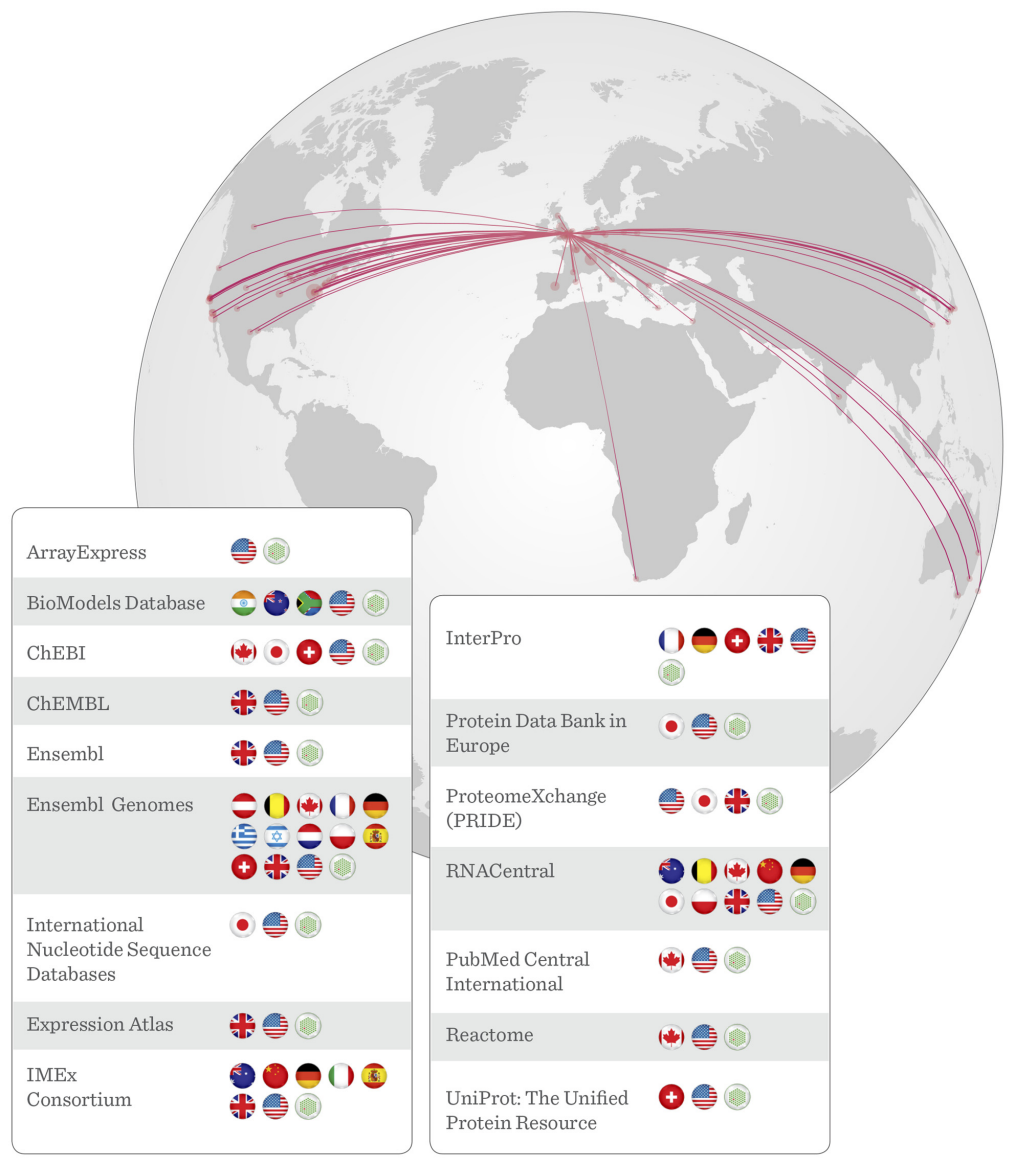
Major database collaborations. Many EMBL-EBI data resources are collaborations with other organizations around the world. These collaborations allow us to share the effort of collecting and serving data to our users and to provide collectively more and better analytical tools. This map visually demonstrates the shared global network that supports open data for the biological sciences.

Our scientific data resources (https://www.ebi.ac.uk/services) include archives, value-added knowledgebases and integrating services for molecular data analysis. With few exceptions, these resources are freely available without restriction. Fifteen of our resources are run as collaborations with partners in 16 countries, in which data or metadata are shared so that users can search for, and find, resource-specific data relevant to their research through any of the partners in the collaboration (Figure 2).

EMBL-EBI is an active partner in the ELIXIR infrastructure (https://www.elixir-europe.org/about-us), which aims to coordinate life-science resources throughout Europe so that they form a single infrastructure. In July 2017, ELIXIR announced the selection of the first set of ELIXIR Core Data Resources (https://www.elixir-europe.org/platforms/data/core-data-resources). These are life science data resources that are of fundamental importance to the worldwide life-science community and to the long-term preservation of biological data (1). EMBL-EBI participated actively in the development of the criteria for selecting ELIXIR Core Resources, and 12 EMBL-EBI resources were included in the initial set of ELIXIR resources: ArrayExpress (2), ChEBI (3), ChEMBL (4), the European Genome-phenome Archive (EGA) (5), the European Nucleotide Archive (ENA) (6), Ensembl (7), Ensembl Genomes (8), Europe PMC (9), IntAct (as part of the IMEx Consortium) (10), InterPro (11), the Protein Data Bank in Europe (PDBe) (12), PRIDE (part of the ProteomeXchange consortium) (13) and UniProt (14). ELIXIR has also compiled a list of archival resources that are recommended for deposition of experimental data. Archival resources are vital to the scientific community for ensuring that experimental data are available for re-use by the world-wide community. Eight EMBL-EBI resources were named as ELIXIR deposition databases: ArrayExpress (2), BioModels (15), EGA (5), ENA (6), IntAct (10), MetaboLights (16), PDBe (12) and PRIDE (13); with four additional databases to be included in the near future: BioSamples (17), BioStudies (18), EVA (19) and Electron Microscopy Data Bank (EMDB) (20).

Archival resources, for example the ENA, BioSamples, PRIDE and Array Express, are the first port of call for data sharing, and store raw experimental data submitted by researchers. They provide a foundation for the knowledgebases such as Ensembl, UniProt and the Expression Atlas (21), which enrich and update annotation and develop tools for access and analysis.

Europe PMC, the data resource for life-science research articles, has developed the SciLite application for displaying text-mined annotations from the community on full text articles (9). These highlights of key biological entities and relationships both aid skim-reading—a particular requirement for database curators—and provide deep anchor points within full-text articles for precise linking between knowledgebase databases and research papers. One of the text-mined entity types is database accession numbers. Over 20 accession number types are mined on a routine basis, providing a further layer of integration, in particular for deposition databases. To improve access to the ‘data behind a paper’, Europe PMC now generates a record in BioStudies (18) for all papers that mention or cite data in community archives in the text, or have supplementary data files or both. This approach allows for connectivity across all the data resources that support a study.

To facilitate the use of publicly available molecular data in R&D pipelines across sectors, we offer programmatic access to almost all of our data resources. Since our last update (19) we have launched several new tools for access, including application program interfaces (APIs) for nucleotide (https://www.ebi.ac.uk/about/news/service-news/new-ena-discovery-api), expression (https://www.ebi.ac.uk/about/news/service-news/restful-rna-seq-analysis-api-v1.2) and protein sequence data resources (22).

We also offer programmatic and web access to about 120 computational tools (https://www.ebi.ac.uk/services/all), and help users bring their own data and store their results for future reference.

## OPEN DATA FOR A GLOBAL COMMUNITY

Open data are a springboard for innovation and discovery in the life sciences. We provide data for open use (https://news.embl.de/lab-matters/1506-open-access/), almost always using the CC-BY (https://creativecommons.org/licenses/by/4.0/), Creative Commons CC0 (https://creativecommons.org/publicdomain/zero/1.0) licenses or their equivalent. There are some restrictions on access for potentially identifiable human data, which requires approval by data access committees. We work closely with data access committees and our partners in ELIXIR and the Global Alliance for Genomics and Health (GA4GH, https://www.ga4gh.org/) to ensure that datasets from individual humans in the EGA are made available only to approved researchers (5).

Most funders have open-access policies that require researchers to deposit research data in publicly available, open-access data resources. Our data resources, used on their own or in conjunction with those managed by other organizations, have become integral to life sciences research, biotechnology and pharmaceutical R&D and, increasingly, to medical and clinical research. Open-access policies enforced by funders, in conjunction with the increasing usage of global public data resources (see below), are putting strain on data resource providers including EMBL-EBI. This is a good problem to have, but the challenges of managing data resources are compounded as demand continues to exceed growth in capacity.

In 2017, a coalition of funders and data providers, including representatives from EMBL-EBI, published a statement that ‘the life sciences should be supported through a coordinated international effort(s) that better ensure long-term sustainability and that appropriately align funding with scientific impact’ (23). We support this effort and will report on progress in future.

## NEW DATA TYPES

### Imaging

Biological knowledge is largely in reference to observations made with the naked eye, and imaging will always be a stanchion of research practice. Recent advances in imaging technologies have generated a range of highly reproducible image types that, like molecular data, are useful as reference data for research projects of all sizes. In 2017, the University of Dundee, EMBL-EBI, the University of Bristol and the University of Cambridge published the Image Data Repository (24): a prototype public repository for imaging data. It is a first step toward addressing the need for open-access, re-usable imaging data.

EMBL-EBI also provides data resources for electron microscopy, which can generate thousands of high-resolution images per experiment. Approximately 20% of the 1000 new data entries in the EMDB in 2016 were from tomography and sub-tomogram averaging experiments (20); of these, 40% were at reported resolutions better than 6Å. The Electron Microscopy Public Image Archive (EMPIAR) includes raw data from three-dimensional (3D) scanning electron microscopy and soft X-ray tomography image data (20). Cryo-EM technology is still in the ‘emergent’ phase, and EMPIAR provides the data and compute needed to develop agreed, ‘gold-standard’ methods and for training in data analysis. Community-led ontology development is underway (25) to enrich EMPIAR metadata (e.g. sample preparation, equipment), which further facilitates the development of methods and standards.

High-resolution 3D imaging has also become a key component of mouse-phenotyping data resources. EMBL-EBI, in collaboration with MRC Harwell and Queen Mary University of London, manages data for the International Mouse Phenotyping Consortium (IMPC). The IMPC catalog offers functionality to detect similarities between mouse data and 7000 human diseases automatically (26). It incorporates high-resolution 3D imaging and automated, computational analysis of these images. All data and images are available via an open-source, web-based resource, without embargo (27).

### Biobanks, biosamples and blood

In 2017, the EGA (5), which is developed jointly with the Centre for Genomic Regulation in Barcelona, entered a partnership with the UK Biobank to store and manage genetic data generated from its samples (https://www.ebi.ac.uk/about/news/press-releases/ukbiobank-genetic-data-ega). The UK BioBank stores biological samples from 500 000 individuals whose phenotypes have also been described. EMBL-EBI’s expertise in data storage and curation improves integration of genetic and phenotypic information from each individual and sample to enhance analysis of research using the biobank.

The BioSamples database (17) links diverse data from over 5 million biological samples, some of which have been used in many different experiments over an extended period of time. The resource now offers sample information from the European Bank for induced pluripotent Stem Cells (EbiSC, https://www.ebisc.org/), the Functional Annotation of ANimal Genomes (https://www.animalgenome.org/community/FAANG/) project and the Human Induced Pluripotent Stem Cell Initiative (http://www.hipsci.org/), among others.

EMBL-EBI provided data coordination for BLUEPRINT, an EU-funded International Human Epigenome Consortium initiative to generate the reference epigenomes of cell types from human blood (28). In 2016, the project released over 1000 datasets representing over 50 primary cell types from healthy individuals, the neoplastic counterparts of those cell types, and other data on patients with type 1 diabetes. The 8000 datasets are freely available through Ensembl (7).

### Emerging data types

The lack of common standards to describe phenotypic data has hampered the exchange and reuse of plant phenotype data, which is extremely diverse. We are part of a standards initiative called MIAPPE: Minimum Information About a Plant Phenotyping Experiment, which has put forward a minimal set of guidelines (29,30) for plant phenotypic experiments that specifies both the content and format of the description. The implementation of MIAPPE standards will ensure metadata is delivered to the public archives in an automated fashion, but new tools are required to make this feasible on a large scale.

## DATA INTEGRATION

The open-source Omics Discovery Index, launched in 2017, provides a single point of access to all public genomics, proteomics, metabolomics and other large-scale datasets in 11 member repositories hosted by six different organizations (31). It addresses interoperability through shared identifiers, rich metadata, ontology-based tools, a flexible exchange system based on an XML format and APIs.

Our BioModels team helped launch PharmML, a flexible format for exchanging computational models in pharmaceutical R&D, in 2016 (15). PharmML is a key component of the Innovative Medicines Initiative-funded DDMoRe model repository (http://repository.ddmore.eu/) (32), which supports inter-organizational collaboration on models to improve the design of cost-effective, reliable clinical trials of new and re-purposed drugs. The Proteomics team, in collaboration with the Proteomics Standards Initiative, has led the development of the proBed and proBAM standard formats, aimed at representing proteogenomics results (https://www.biorxiv.org/content/early/2017/06/20/152579). The idea is that these formats are compatible with the original BED and SAM/BAM genomics formats, streamlining the integration of proteomics and genomics information.

RNAcentral (33) is a comprehensive database of non-coding RNA sequences (ncRNA) that represents all types of ncRNA from a broad range of organisms. It provides a unified entry point for the ncRNA data from 25 specialized resources, including the recently integrated Ensembl (7), the HUGO Gene Nomenclature Committee (34) and FlyBase (35) ncRNA datasets. In the last year RNAcentral has seen an increase of over one million new ncRNA sequences.

In PDBe, a new toolkit integrates the cellular structure and molecular structure with other forms of bioinformatics information, translating between existing segmentation file formats and EMDB-SFF, the format that supports structured biological annotations. The toolkit complements our extended SIFTS pipeline, which maps UniProt and PDB information (12).

### Ontologies

In the ontology space, we are working to align our data resources semantically to improve integration, search, analysis and visualization. We build tools for semantic enrichment to enable interoperability, and to expand the possibilities for querying biomedical data, for example enriching BioSolr with ontology content. The success of this approach can be seen in the Open Targets platform, which is designed to help pharmaceutical researchers access data relevant to drug–target validation intuitively (36).

We updated and relaunched several ontology services in 2017. The Ontology Lookup Service (OLS), a repository for biomedical ontologies, provides a single interface for searching across numerous ontologies (37). It includes the Human Phenotype Ontology (38) and the Experimental Factor Ontology (https://www.ebi.ac.uk/efo/), which is cross-referenced to 25 additional ontologies. The OLS allows users to map a term to different ontologies using the new Zooma tool (https://www.ebi.ac.uk/spot/zooma/). We are also participating in a Pistoia Alliance project: the Ontology Mapping Service (https://www.ebi.ac.uk/spot/oxo/), which is mapping disease terms between ontologies and facilitating industry access to public molecular data resources.

## DATA GROWTH

**Figure 3.**
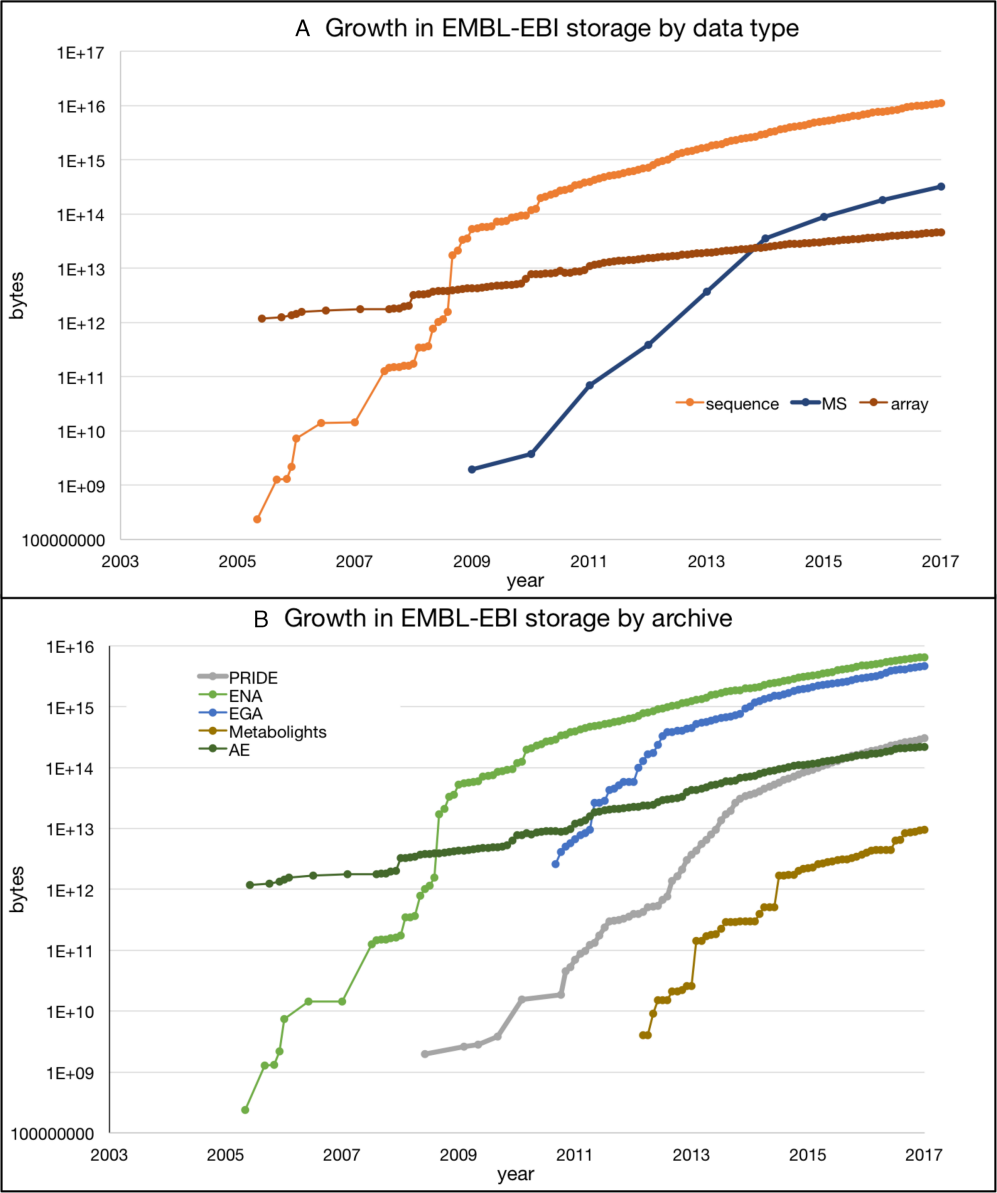
(**A**) Data accumulation at EMBL-EBI by data type: nucleotide sequence, mass spectrometry and microarray; (**B**) Data accumulation by archive: Proteomics identifications (PRIDE) (13), EGA (5), ArrayExpress (AE) (2), European Nucleotide Archive (ENA) (6) and Metabolights (16). The *Y*-axis for both charts is logarithmic, so growth in all data types is exponential. In all data resources shown here, growth rates are predicted to continue increasing, with notable sustained exponential growth in PRIDE, the EGA and MetaboLights. All have doubling times of around 12 months.

Our public molecular archives continue to grow at an exponential rate, and there is little reason to believe this will slow down (Table 1). Raw nucleotide sequence continues to dominate: the ENA added 60 million new assembled/annotated sequences and 1.5 × 10^15^ base pairs of read data in 2016 alone. Compression techniques such as CRAM have mitigated the rate of increase in submission volume, but growth is still exponential for nucleotide data. Figure 3 shows the growth of EMBL-EBI data by platform.

**Table 1.** Growth in selected EMBL-EBI resources 2015 to 2016

Platform	2015	2016	Percent increase
Protein families, motifs and domains (in InterPro)	28 678	29 700	4
Macromolecular structures	114 691	125 463	9
Gene expression assays	1.9 million	2.2 million	16
PRIDE database	1523 datasets	1950 datasets	28
Protein sequences	55.2 million	71.0 million	29
Nucleotide sequence data	4.52 PB	5.91 PB	31
Genomes—all species and strains, including prokaryotes.	30 674	42 529	39
Metagenomic sample datasets	8000	Over 75 000	838

MetaboLights (not shown) became the recommended metabolomics repository for several major journals, so we anticipate significant future growth (16).

Year on year, we see a 40–50% increase in demand for storage. As of September 2017, our capacity was 120 Petabytes; this figure is expected to rise to exabyte scale by 2022. Our current approach to storage is a blend of fastest (SSD), fast-access HD, normal HD and tape backup. To maximize efficiency and resiliency, and to reduce the amount of duplication required, we maintain a geographically distributed Object Store. This allows us to use less storage to maintain reliable backups, reducing the rate at which we need to procure new disk space. Other storage solutions range from general-purpose scale-out such as Netapp and Isilion (which grow to multi petabyte) to high-performance systems like Lustre, Spectrum Scale and our bespoke object tape archive.

The ENA team has worked extensively on pathogen data services and coordination, launching 15 pathogen-related ‘data hubs’ and a pathogen website (https://www.ebi.ac.uk/ena/submit/pathogen-data). The number of bacterial genomes available through Ensembl Bacteria increased to nearly 40 000, the number of fungal genomes in Ensembl Fungi to over 700, and the number of protist genomes in Ensembl Protists to almost 200 (8).

EBI Metagenomics (39) is a freely available hub for the archiving, analysis and exploration of metagenomic datasets. It provides rich functional and taxonomic analyses of user-submitted sequences, as well as analysis of publicly available metagenomic datasets held within the European Nucleotide Archive. Publicly available metagenomics data grew 11-fold in 2016 and, in collaboration with its US counterpart MG-RAST, launched the Metagenomics Exchange to promote data exchange between the two platforms. In 2017, EBI Metagenomics released a new service for metagenomic assembly that allows users to run analyses on its vast datasets in very short timeframes.

Our gene expression resources now hold data from over 70 000 experiments and 2.2 million assays from 30 organisms, with the RNA-seq studies in our Baseline Expression Atlas including data from both large-scale studies such as BLUEPRINT and smaller-scale proteomics experiments. As of August 2017, we have curated over 3000 transcriptomics experiments and comprising over 100 000 assays. These assays included over 500 RNA-seq experiments, over 8000 differential comparisons and over 700 plant experiments (21).

In the proteomics domain, submissions to the PRIDE database (as part of ProteomeXchange Consortium) (13) are growing steadily (1950 datasets submitted in 2016; over 1950 submitted by 1 October 2017). PRIDE has become the third largest EMBL-EBI archive in terms of data volume (Figure 3B). This facilitates the reuse of public proteomics data (40), as demonstrated by the increased volume of data downloaded from the resource (∼250 Terabytes in 2016).

As of August 2017 the number of novel, annotated chemical entities in SureChEMBL has reached 19.1 million (41). The resource is growing at a rate of around 80 000 novel chemicals per month curated from roughly 50 relevant bioactivity data. In order to extract such detailed information, we have collaborated with the BindingDB Group at the University of California, San Diego (42) and separately with the NIH-funded Illuminating the Druggable Genome project to incorporate patent-derived bioactivity data into ChEMBL (4). These additional entries, which involve a ‘curation step’ by human experts, cover druggable protein families for which there is little or no bioactivity data in the published literature and demonstrate that patents can be a source of novel scientific information if mined carefully.

## DATA ANALYSIS

The ENA’s new Discovery API and toolkit allows for the rapid deployment of data presentation websites and launch of a cloud-based computational analysis environment. Offered as a RESTful interface, the service also allows comprehensive search against the entire ENA content and supports logical searches across fields from distinct data types (6).

Ensembl’s new Advanced Search tool allows comprehensive, fast queries of genomic annotation of over 80 million gene models and associated data from ∼20 000 species (7). It also retrieves variation and expression data from the European Variation Archive and Expression Atlas (19,21).

Our new RESTful RNA-seq Analysis API applies ontology-based search to expression data (https://www.ebi.ac.uk/fg/rnaseq/api/). In addition, a new pipeline automatically aligns public RNA-sequence data against the genome sequence. Users can now visualize expression data from over 1000 distinct experiments within Ensembl Plants and Ensembl Fungi (43).

We launched a new tool for integrating large-scale molecular data with known annotations. The new Proteins REST API provides access to key biological data from UniProt and large-scale studies data mapped to UniProt (https://www.ebi.ac.uk/proteins/api/). It also serves as a bridge between genomic and protein data, enabling users to retrieve genome coordinates for protein sequences.

New chemistry web services, based on RDkit, allow users to perform more complex queries and combine data in ChEMBL. A Solr-based search supports those accessing ChEMBL and UniChem programmatically through APIs (4).

## CLOUD-BASED COLLABORATION

Cloud analysis will become increasingly important in future as datasets become too large to move around, and as analytical requirements become too large for individual institutions to support large computational infrastructures.

Embassy Cloud, our infrastructure-as-a-service, is based on the OpenStack cloud platform (http://www.embassycloud.org/about/). It features private, secure ‘tenancies’ hosted within our data centers, providing users with direct access to datasets and services and negating the need to download large data resources before undertaking analyses. Embassy Cloud is currently used by external research consortia in collaboration with EMBL-EBI teams. It includes 6000 cores, 40 terabytes of RAM, 50 terabytes of SSD fast scratch space, two petabytes of Network File System (NFS) and two petabytes of ‘object store’ storage.

Through the European Open Science Cloud, we are making our data services more accessible in external cloud services. This means that users can use public cloud or local ELIXIR cloud services to carry out their computation (https://eoscpilot.eu/).

## TRAINING

Our Training Program continues to offer an increasing number of courses at EMBL-EBI in Hinxton and at host sites throughout the world, as well as online training and a new webinar series (https://www.ebi.ac.uk/training/online/). These activities are designed to help professionals exploit data from new and emerging technologies, which can be challenging in a time of rapid technological change and scientific advancement.

We consult closely with the community to identify gaps in training provision, and seek to address them across sectors. Since our last update we have developed a competency-based approach to identifying training needs (44) and have applied these in several contexts, including research infrastructure management and operation and use of high-end compute for biomolecular research.

Our Training program is collaborating with nine research institutes in Latin America to launch the CABANA project. Funded by the Research Councils UK, CABANA aims to speed up the implementation of data-driven biology in Latin America. It will include research secondments, ‘train-the-trainer’ workshops, short courses and e-learning resources. These activities will empower researchers to use bioinformatics tools better and contribute more data to bioinformatics databases. One of the program’s most important objectives is to strengthen existing research networks in the area.

## LOOKING AHEAD

To serve our diverse user community, we are focusing on improving efficiency by enhancing interactions among our data services. This will improve search results and ensure that users can find everything relevant to their research that is in our data resources. We are also working to provide a single, user-friendly starting point for data deposition that will streamline data submission.

Molecular data has a huge potential to create clinically useful treatments and diagnostics. EMBL-EBI’s data resources are use in both basic and translational research and we are working to build bridges between biological information and clinical data to develop medically relevant data resources and infrastructures. Specifically, within the Global Alliance for Genomics and Health driver projects we will contribute to enabling genomic data sharing in clinical medicine by developing, testing and advocating for the uptake of standards, tools, frameworks and best practice.

Our data coordination activities will continue to intensify, notably in the context of the Human Cell Atlas data-coordination platform (BioRxiv: https://doi.org/10.1101/121202). To enable research in this single-cell genomics-intensive initiative we will help establish best-practice computational methods and their deployment in the cloud, taking a user-centered approach. We will work with our partners to launch a prototype platform that is carefully engineered before the experimental work is undertaken. This will enable laboratories of all sizes to access high-value datasets and deploy pipelines easily to carry out advanced, large-scale analyses.

Growth in data volumes and new resources for emerging or previously underserved data types, such as imaging, will require sustained commitment. In Europe and worldwide, EMBL-EBI and other scientific data providers are addressing the challenges that arise from this growth through collaboration and cooperation (see Figure 2). The continued interaction between EMBL-EBI resources and their partners over the past decades demonstrates how collaboration allows public-sector data providers to share costs while improving services for an ever-growing and diversifying community of users.

The ELIXIR research infrastructure in Europe is actively working to share data resources and expertise throughout Europe. The importance of such exchange has long been recognized, and there is strong support for the establishment of more formal worldwide mechanisms to coordinate the provision of data resources for biomedical and biological research that could, for example, ensure more efficient, longer-term financial support of data resources through collaboration among funders (23). EMBL-EBI is participating actively in this effort.

Bioimaging, single-cell and biobanking data offer exciting opportunities and challenges for public research infrastructure. The bottleneck will be, as ever, data analysis. We will continue to create, in collaboration with academic and commercial researchers, bespoke bioinformatics algorithms and analysis pipelines to facilitate discovery and development. We will also continue to foster collaboration between basic, curiosity-driven research and clinical, healthcare and commercial R&D in all life-science domains. With continued support and commitment from our collaborators and funders, we look forward to supporting the community in pushing these frontiers of science.
